# Comparison of the Effects of High Hydrostatic Pressure and Pasteurization on Quality of Milk during Storage

**DOI:** 10.3390/foods11182837

**Published:** 2022-09-14

**Authors:** Tongtong Yu, Xiaojun Zhang, Ruoyi Feng, Caiyun Wang, Xiaoyu Wang, Yongtao Wang

**Affiliations:** 1 College of Food Science and Nutritional Engineering, China Agricultural University, Beijing 100083, China; 2 Inner Mongolia Dairy Technology Research Institute Co., Ltd., Hohhot 010000, China; 3 Inner Mongolia Yili Industrial Group Co., Ltd., Hohhot 010110, China

**Keywords:** high hydrostatic pressure, pasteurization, physicochemical characteristics, nutrient content, sensory evaluation

## Abstract

High hydrostatic pressure (HHP, 600 MPa/15 min), pasteurization (72 °C/15 s) and pasteurization-HHP (72 °C/15 s + 600 MPa/15 min) processing of milk were comparatively evaluated by examining their effects on microorganisms and quality during 30 days of storage at 4 °C. The counts of total aerobic bacteria in HHP-treated milk were less than 2.22 lgCFU/mL during storage, while they exceeded 5.00 lgCFU/mL in other treated milk. Although HHP changed the color, it had more advantages in maintaining the nutrient (fat, calcium and β-lactoglobulin) properties of milk during storage. Moreover, the viscosity and particle size of HHP-treated milk were more similar to the untreated milk during storage. However, consumer habits towards heat-treated milk have led to poor acceptance of HHP-treated milk, resulting in a low sensory score. In sum, compared with pasteurization- and pasteurization-HHP-treated milk, HHP-treated milk showed longer shelf life and better nutritional quality, but lower sensory acceptance.

## 1. Introduction

Milk is one of the most popular and widely consumed dairy products in the world, is rich in protein, fat, carbohydrates and a variety of immunologically active factors and can be easily digested and absorbed by the human body [1,2]. However, the rich nutrition of milk may easily cause microbial contamination, which reduces the quality and shelf life of the product [3]. Heat treatment is a traditional method to ensure the quality and safety of milk, mainly including ultra-high temperature (UHT) instant inactivation (135–140 °C, 2–10 s) and pasteurization (72 °C, 15 s, or 63 °C, 30 min) [4]. The low temperature used in pasteurized milk can minimize the loss of the original quality of the milk, but the effect of killing heat-resistant bacteria and inactivating enzyme activity is limited, leading to the problem of short shelf life [5,6]. At the same time, pasteurized milk must be stored, transported and sold at 2–6 °C, which limits the market to a certain extent. In contrast, UHT milk is convenient to store, transport and sell at room temperature, which greatly reduces production costs. However, excessive intensity of heat treatment causes irreversible damage to the original quality of the milk, and even produces harmful substances, as well as adversely affecting the flavor of the milk [7,8].

High hydrostatic pressure (HHP), a mature non-thermal processing technology, has been widely used in the food industry. Its earliest application in the food field was sterilization [3,9]. HPP leads to microbiological inactivation due to two reasons: cell injury and protein denaturation. During HHP treatment, the denaturation of enzymes, which are important for the metabolism of bacteria, occurs. In bacterial cells, due to the change in osmotic pressure, membrane structure might be altered and injured, causing leakage of the cell contents and cell death [10,11]. In addition, HHP is considered to be a physical process, in that pressure destroys non-covalent bonds, such as hydrogen bonds, disulfide bonds, and hydrophobic bonds, in the three-dimensional structure of biological macromolecules, but has little effect on covalent bonds. In consequence, HHP has almost no influence on small molecules, such as vitamins, flavors and pigments [12,13,14]. Therefore, compared with traditional heat treatment, HHP can retain the original color, aroma and taste of food to the greatest extent. This advantage also reduces the use of food additives and decreases the risk of chemical substances, in line with the development trend towards clean labels, which is of great significance today when minimal processing is advocated [9,15].

Although there have been numerous reports on the application of HHP in milk, they have mostly focused on a single aspect, such as research on the inactivation effect of HHP on specific microorganisms in milk or the denaturation of proteins under HHP [10,16,17,18]. The research on the quality of milk, as it concerns commercial production, is not comprehensive and systematic.

In this study, the effects of HHP, pasteurization and pasteurization-HHP treatments on microorganisms, physicochemical characteristics, nutrients and the sensory quality of milk during storage were investigated to systematically evaluate the feasibility of HHP in milk production.

## 2. Materials and Methods

### 2.1. Raw Materials and Chemicals

The raw milk used in this study was homogenized at 55 °C/25 MPa for commercial production provided by Inner Mongolia Yili Industrial Group Co., Ltd. Plate counting medium and Bengal red medium were purchased from Beijing Solarbio Technology Co., Ltd. (Beijing, China). Methanol was purchased from Merck (Kenilworth, NJ, USA). Other chemicals were purchased from Beijing Solarbio Technology Co., Ltd. (Bejing, China).

### 2.2. HHP, Pasteurization and Pasteurization-HHP Treatments of Milk

For the HHP treatments, polyethylene terephthalate bottles of 100 mL capacity with screw-cup closures were filled with milk and placed into the vessel for processing. HHP treatments were carried out using a hydrostatic pressurization unit (CQC30L-600, Beijing Suyuan Zhongtian Technology Co., Ltd., Beijing, China) with a capacity of 30 L at ambient temperature (~25 °C). The pressurization rate was about 120 MPa/min and the depressurization was immediate (<3 s). Distilled water was used as the pressure-transmitting fluid. The treatment time reported in this study did not include the pressure-increase time and pressure-release time. To determine the appropriate treatment conditions, the milk was treated with HHP under 15 conditions, including 3 different pressures (200, 400, and 600 MPa) and 5 different processing times (2.5, 5, 7.5, 10, and 15 min), and the group of conditions with the best inactivation effect was selected for subsequent storage experiments (microbiological analysis was performed immediately after treatment, see Section 2.4 for the method).

According to Liu et al. [19] with minor modifications, pasteurization-treated milk was processed (72 °C/15 s) in a pilot scale pasteurizer with a tubular heat exchanger (Armfield FT74, HTST/UHT Processing Unit, Hampshire, England) and filled under aseptic conditions. Pasteurization-HHP-treated milk was obtained after pasteurization followed by HHP, according to the above method.

### 2.3. Storage Conditions

The samples were stored in a 4 ± 2 °C refrigerator away from light. Analyses were carried out after 0, 3, 7, 11, 15, 22 and 30 days of storage. 

### 2.4. Microbial Analysis

To count viable microorganisms in milk, the total plate count method was used according to Wang et al. [20]. An untreated or treated sample was serially diluted with sterile 0.9% NaCl solution, and 1.0 mL of each dilution was plated into duplicate plates of appropriate agar. Nutrient agar (Beijing Solarbio Technology Co., Ltd., Beijing, China) was used for counting the viable TAB cells after incubation at 37 °C for 48 ± 2 h. Rose bengal agar (Beijing Solarbio Technology Co., Ltd., Beijing, China) was used for counting cells of the viable yeasts and molds (Y&M) after incubation at 27 °C for 72–120 h. After incubation, the colonies were counted.

### 2.5. Physicochemical Characteristics Analysis

The pH value was measured at 25 °C with a Thermo Orion 868 pH meter (Thermo Fisher Scientific, Inc., Waltham, MA, USA). The turbidity value was measured at 25 °C with WGZ-2000 Turbidity meter (Shanghai Youke Instruments Co., Ltd., Shanghai, China) after diluting the sample 200 times with ultrapure water.

Color assessment was conducted at 25 ± 2 °C using a color measurement spectrophotometer (HunterLab ColorQuest XE, Hunter Associates Laboratory, Inc., Reston, VA, USA) in the reflectance mode. Color was expressed in L*, a* and b* values. In addition, total color difference (Δ*E*) was calculated using the following equation, where *L*_0_*, a_0_* and b_0_* are the control values for untreated samples:

Δ*E* = [(L* − L_0_*)2 + (a* − a_0_*)2 + (b* − b_0_*)2]^1/2^ (1)

### 2.6. Determination of Protein, Fat, Calcium, Ash and β-lactoglobulin (β-LG) Content

The protein content was determined by the Kjeldahl method, the fat content was determined by the alkaline hydrolysis method, the calcium content was determined by inductively coupled plasma emission spectrometry and the ash content was determined by muffle furnace referred to Chinese Standard GB 5009.5-2016, GB 5009.6-2016, GB 5009.268 and GB 5509.4-2016, respectively [21,22,23,24].

According to Wazed et al. [25], with some modifications, the content of β-LG in milk was determined by the enzyme-linked immunosorbent assay (ELISA) kit (SEB023Bo, Cloud-Clone Corp., Wuhan, China). The sample was centrifuged at 10,000 rpm for 15 min at 4 °C, and the supernatant was diluted 1000 times with PBS buffer solution with pH 7.0 before measurement. Then the substrate solution was added to the mixture for color development at 37 °C in the dark. The absorbance was measured at 450 nm using a spectrophotometer (UV-726, Shimadzu, Shanghai, China) after the stop solution was added to the system. The content of β-LG was calculated according to the standard curve (Y = 0.6595x + 0.1596, R^2^ = 0.9922).

### 2.7. Quantification of Alkaline Phosphatase Activity

The alkaline phosphatase activity was measured according to Liao et al. [26] with slight modifications. A quantity of 0.500 g disodium phosphate was dissolved in 10 mL distilled water, then, 25 mL of phosphate buffer, 2 drops of 2,6-dichloroquinone chlorimide solution and 1 drop of copper sulfate solution were added. After standing, 3 mL n-butanol was added to the mixture, the components of water phase components were released after layering and the substrate developer buffer was obtained by diluting the rest to 500 mL and storing at 4 °C for use. Milk was diluted 80-fold with distilled water, and 1 mL of diluted sample was mixed with 10 mL of substrate developer buffer and incubated in a water bath at 40 °C for 15 min. After that, 0.1 mL of 2,6-dichloroquinone chlorimide solution and 2 drops of copper sulfate solution were added to the mixture and incubated at 40 °C for 5 min. Then, the mixture was mixed with 20 mL n-butanol and centrifuged using 3000 rpm/min for 5 min. The absorbance was measured at 655 nm using a spectrophotometer (UV-726, Shimadzu, Shanghai, China) and the alkaline phosphatase activity was calculated according to the standard curve (Y = 0.0315x + 0.0041, R^2^ = 0.9998).

### 2.8. Quantification of Furosine

Referring to Zhang et al. [27], with slight modifications, quantification of furosine was carried out by the external standard with High Performance Liquid Chromatography (HPLC). A quantity of 5 mL of milk was mixed and sealed with 6 mL HCl (10.6 mol/L) and heated at 110 °C for 18 h. Then, 1 mL of hydrolysate was added to 5 mL ammonium acetate solution (6 g/L), and the mixture was passed through a 0.22 μm cellulose nitrate membrane for HPLC. The HPLC system (Knauer Co., Ltd., Berlin, Germany) was composed of a K-1001 pump (Knauer Co., Ltd., Berlin, Germany), connected to a refractive index detector (RI-2301, Knauer Co., Ltd., Berlin, Germany), and a 20 μL injection loop. The column was Agilent Eclipse SB-C18 (4.6 × 250 mm i.d, 3.5 μm particle size). The mobile phase A was methanol and mobile phase B was 0.1% trifluoroacetic acid. The flow rate was 0.8 mL/min at 32 °C. The detection wavelength was 280 nm.

### 2.9. Viscosity Analysis

The viscosity of milk was measured using a TA-1000 rheometer (American TA Instruments Co., Ltd., New Castle, DE, USA). Viscosity measurement of concentrates was performed at 25 °C to eliminate temperature differences between samples. Samples were sheared using an increasing order from 0.5 s-1 to 100 s-1. To ensure stable readings, the time interval of viscosity recordings was 1 min for each shear rate. 

### 2.10. Particle Size Measurement

The distribution of particle size and protein particle size of milk was measured by an LS 230 Laser scattering particle size analyzer (Beckman Coulter, Inc., Brea, CA, USA). The particle size distribution curves were obtained depending on Mie theory. The results were expressed as follows: D(4,3) = volume mean diameter; D(2,3) = surface area mean diameter.

### 2.11. Sensory Evaluation

Sensory evaluation was conducted by the method provided by Inner Mongolia Yili Industrial Group Co., Ltd. (Hohhot, China). A total of 15 assessors (8 females and 7 males) were invited to conduct the sensory evaluation of milk. All assessors were non-smokers and screened for recognition and thresholds of sweet, acidic, salt, bitter and umami taste, as well as the ability to perform sensory tasks and interact in discussions of sensory attributes.

Samples were scored in terms of taste (the score ranged from 0 to 40), color (the score ranged from 0 to 20), organizational status (the score ranged from 0 to 20) and flavors (the score ranged from 0 to 20), as listed in Supplementary Materials, Table S1. The assessors were familiar with the sensory evaluation of dairy products and before each task they participated in one training session. During this session, the assessors became familiar with the products, the vocabulary and the exact evaluation procedure. The color and texture of the samples were observed under natural light conditions, and the taste was evaluated after rinsing with warm water. These sensory qualities were evaluated on the processing day and at every storage interval until the end of the shelf life during storage at 4 °C. The scores were collected and the average values and total scores were calculated.

### 2.12. Statistical Analysis

Principal Component Analysis (PCA) is commonly used for data dimensionality reduction. In order to differentiate the quality of untreated, HHP-treated, pasteurization-treated and pasteurization-HHP-treated milk from an overall perspective, the raw data of physicochemical characteristics, nutrient content and sensory quality were subjected to PCA analysis. PCA was supported by MetaboAnalyst, which was accessible at http://www.metaboanalyst.ca, according to Lwin et al. [28], and the analysis was performed using the prcomp package.

Samples processed with different treatments included three replicates, and all data was expressed as the average of the replicates. The data were analyzed using the Statistical Program for Social Sciences (SPSS 23.0, Chicago, IL, USA) software for analysis of variance and Duncan’s test. The significance was established at *p* < 0.05. Graphics were produced using the Origin Pro 2018 software (OriginLab, Northampton, MA, USA).

## 3. Results and Discussion

### 3.1. Microbiological Analysis

The number of surviving cells of milk after treatments was determined by monitoring the TAB and Y&M counts. As shown in Table 1, the inactivation effect of HHP was enhanced with the extension of pressure and processing time. The initial count of TAB in milk was 4.19 lgCFU/mL and this was significantly reduced to 3.84, 2.85, 2.40 and 1.12 lgCFU/mL after 400 MPa/2.5 min, 400 MPa/15 min, 500 MPa/15 min and 600 MPa/15 min treatment, respectively. This result was consistent with the results reported by Liepa et al. [16], who found that the inactivation effect of skimmed milk processed with different treatments was in the following order: 250 MPa/3 min < 400 MPa/3 min < 400 MPa/15 min < 550 MPa/3 min. Additionally, Liu et al. [19] reported that HHP treatment at 600 MPa (5 min, 45 °C) reduced the count of TAB in milk by 3.00 lgCFU/mL, which confirmed that HHP performed an outstanding inactivation effect. Meanwhile, the initial count of Y&M was 1.82 lgCFU/mL and was not immediately detected after 400 MPa/5 min treatment. Hai-Hong et al. [29] also reported that Y&M can be inactivated under a pressure of 200–400 MPa. Based on the above results, 600 MPa/15 min was chosen as the HHP treatment for subsequent storage experiments.

**Figure 1:**
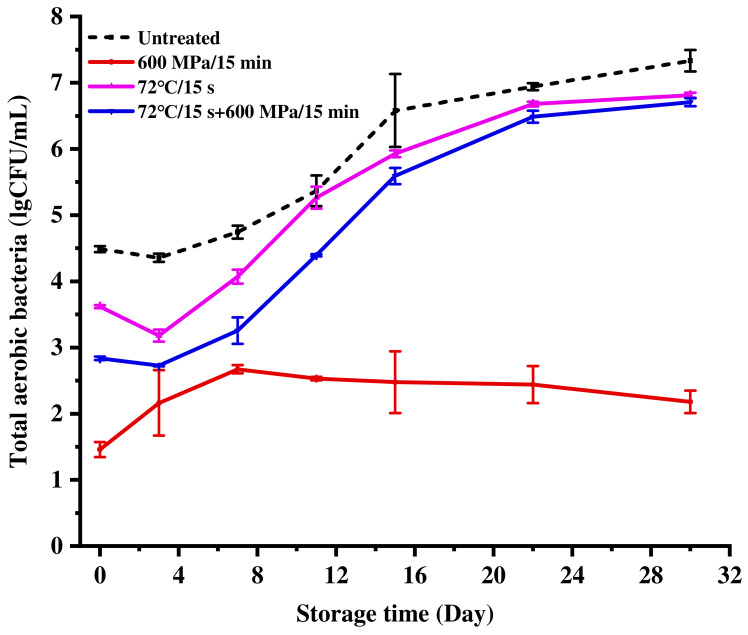
Changes in the number of total aerobic bacteria (TAB) of milk processed with HHP (600 MPa/15 min), pasteurization (72 °C/15 s) and pasteurization-HHP (72 °C/15 s + 600 MPa/15 min) during storage at 4 °C.

As shown in Figure 1, the counts of TAB reduced to 1.45, 3.61 and 2.83 lgCFU/mL after HHP (600 MPa/15 min), pasteurization (72 °C/15 s), and pasteurization-HHP (72 °C/15 s + 600 MPa/15 min) treatments, respectively. During the refrigerated storage of 30 days, the counts of TAB increased to 7.33, 2.22, 6.81 and 6.70 lgCFU/mL in untreated, HHP, pasteurization and pasteurization-HHP milk from 4.49, 1.46, 3.62 and 2.83 lgCFU/mL, respectively. This showed that the count of TAB in HHP-treated milk maintained a low level during 30 days of storage, which met the requirements of Chinese Standard GB 19645-2010, in which TAB count should be less than 5 lgCFU/mL. However, the counts of TAB in pasteurization and pasteurization-HHP milk exceeded the standard up to 11 days (5.26 lgCFU/mL) and 15 days (5.59 lgCFU/mL). Therefore, these results demonstrated that only HHP-treated milk exhibited a better microbiological stability during storage. Similarly, Stratakos et al. [3] found that the counts of TAB in milk were kept below 4.00 lgCFU/mL after 600 MPa/3 min treatment during 14 days of storage at 4 °C.

Curiously, pasteurization-HHP treatment did not perform as well as HHP treatment in inactivation effect. One of the mechanisms that bacteria have developed to survive in unfavorable conditions is the ability to respond to stress situations. Aertsen et al. [30] provided the evidence of heat shock-induced HHP resistance in *E. coli*, as well as a clue as to the mechanism underlying this induced resistance. They found that heat shock protein production upon temperature upshift rapidly occurred, and clearly demonstrated heat shock-induced pressure resistance in *E. coli strain MG1655*. These results may have important consequences for the use of HHP for food preservation, particularly in combination with mild heat treatment. It is speculated that pasteurization, as a mild method, may cause microbial stress response and enhance microbial resistance to HHP adversity, thus, affecting the inactivation effect [31,32].

### 3.2. Physicochemical Characteristics

#### 3.2.1. pH

As shown in Table 2, there was no significant difference in pH between HHP-treated and untreated milk at day 0, while the pH significantly increased to 6.68 and 6.69 after pasteurization and pasteurization-HHP treatment, respectively (*p* < 0.05). This result was in accord with that found by Kim et al. [33] in milk processed at 600 MPa/10 min. The increase of pH in pasteurization- and pasteurization-HHP-treated milk might result from protein denaturation, lactose isomerization or homeostasis of calcium ions caused by heat treatment [34].

HHP-treated samples showed a slight, but not significant, reduction in pH during storage (*p* > 0.05). However, after 30 days of storage, the pH of pasteurization- and pasteurization-HHP-treated milk significantly reduced to 6.18 and 6.39 (*p* < 0.05), respectively, which might have been due to milk spoilage and increased acidity caused by microbial growth [35,36]. This result was consistent with the result shown in Figure 1. The counts of TAB in HHP-treated milk remained stable, resulting in insignificant changes in pH, while the continuous increase of TAB in pasteurization- and pasteurization-HHP-treated milk caused an obvious reduction in pH.

#### 3.2.2. Turbidity

As shown in Table 2, there was a slight, but not significant, difference (*p* > 0.05) in turbidity between pasteurization-treated and untreated milk at day 0. In contrast, the turbidities of milk after HHP and pasteurization-HHP treatments were significantly reduced to 326.10 and 325.45 NTU (*p* < 0.05), respectively. Orlien et al. [37] also found that treatment of skimmed milk with HHP at pressures exceeding 300~400 MPa would lead to reduced turbidity of the milk. Milk turbidity is mainly related to the aggregation and dissociation of casein micelles [38,39,40]. The casein micelles are composed of casein molecules, colloidal calcium phosphate, and water molecules dispersed in the whey serum and are the main light-scattering particles in milk [37,41]. The HHP treatment could decompose the casein micelles into submicelles, and the rearrangement and compression of the submicelles could decrease the particle size, changing the reflectance of the milk, leading to the reduction in turbidity of milk [40,42,43]. Moreover, there was no significant difference in turbidity between HHP- and pasteurization-HHP-treated milk observed at day 0, and all treated milk showed a slight, but not significant, fluctuation in turbidity during storage (*p* > 0.05) (Table 2).

#### 3.2.3. Color

Color differences among HHP-, pasteurization- and pasteurization-HHP-treated milk were determined after processing and during storage. As shown in Table 2, pasteurization treatment exhibited no significant (*p* > 0.05) effects while noticeable reduction was observed in L*, a* and b* values of HHP- and pasteurization-HHP-treated milk in comparison to the untreated sample (*p* < 0.05). The changes in a* and b* values, as a result of high pressure, differed depending on the conditions of processing and the type of milk. Stratakos et al. [3] reported that a* values were significantly decreased after HHP treatment, while another study on ewe milk, subjected to HHP treatment, yielded a statistically significant increase in a* and b* values. [44].

Values in the same line with different superscript lowercase letters indicate significant changes of one sample during storage (*p* < 0.05). However, the L* value was the parameter that was most severely affected by HPP treatments, which was consistent with the results of our study. The decrease in L* value of milk caused by HHP treatment was mainly due to the decomposition of casein micelles in milk under pressure so that the protease could use more surface area to hydrolyze casein, increasing light transmittance [45]. The Δ*E* value is an indicator of the total color difference between different samples; if Δ*E* >2, it is generally believed that human vision can distinguish the difference in color [20]. The Δ*E* value of HHP-, pasteurization-HHP- and pasteurization-treated milk were 4.24, 2.52 and 0.08, respectively, and, in consequence, in the sensory evaluation (see Section 3.7 below), the color score of HHP- and pasteurization-HHP-treated milk was significantly reduced (*p* < 0.05).

During 30 days of storage, the a* and b* values of all treated samples tended to increase, which could be related to the pigment produced by the Maillard reaction. As shown in Figure S1, the content of furosine, the product of the Maillard reaction, increased significantly, indicating that the Maillard reaction occurred during storage. Consequently, the Δ*E* value of HHP-, pasteurization- and pasteurization-HHP-treated milk increased from 4.24, 0.08 and 2.52 to 7.72, 3.22 and 8.88, respectively. This result indicated that the color quality of milk continued to deteriorate during storage, which could be responsible for the decrease in the color score in the sensory evaluation (Table 4).

### 3.3. Nutrient Content

The protein content (3.39 g/100 g) and ash content (0.70 g/100 g) were not significantly different after treatments and were, for all treatments, constant during 30 days of storage at 4 °C (Figure S2).

#### 3.3.1. Fat

**Figure 2:**
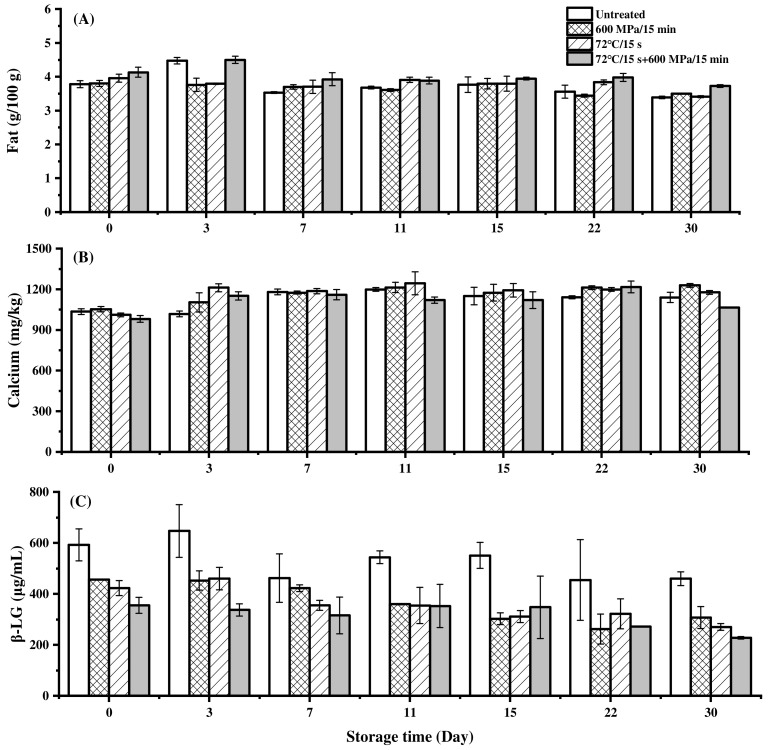
Changes in nutrients content of milk processed with HHP (600 MPa/15 min), pasteurization (72 °C/15 s) and pasteurization-HHP (72 °C/15 s + 600 MPa/15 min) during storage at 4 °C ((**A**) Fat; (**B**) Calcium; (**C**) β-LG).

The fat in milk is an important source of human nutrition. At the same time, milk fat is natural and can dissolve a large number of fat-soluble vitamins, and is easily digested and absorbed by the human body [46,47,48]. As shown in Figure 2A, after HHP, pasteurization and pasteurization-HHP treatments, the fat of milk did not decrease, and the content remained at 3.80, 3.95 and 4.13 g/100 g, which met the requirements of Chinese Drink Standard GB 19645–2010, in which fat should be higher than 3.1 g/100 g.

During storage, the fat content of HHP-, pasteurization- and pasteurization-HHP- treatment milk decreased from 3.80, 3.95 and 4.13 g/100 g to 3.50, 3.42 and 3.73 g/100 g, respectively. The reduction of fat in HHP-treated milk was less than that in pasteurization- and pasteurization-HHP-treated milk, which indicated that HHP treatment could better maintain the fat content during storage. Associated with Figure 1, the counts of TAB in HHP-treated milk maintained a low level during 30 days of storage. In contrast, there was a large increase in the counts of TAB in pasteurization-HHP- and pasteurization-treated milk. It is reasonable to speculate that the fat consumed by microorganisms was the main reason for the decrease in milk fat content.

#### 3.3.2. Calcium

Calcium is the most important and abundant mineral element in the human body, playing an important role in regulating life activities of the body [49]. As shown in Figure 2B, compared to the untreated milk, HHP treatment significantly increased the calcium content (*p* < 0.05), from 1025.00 mg/kg to 1052.75 mg/kg. Nassar et al. [50] also reported that HHP treatment (25 °C, 200/400/500 MPa, 25 min) increased the calcium content in caprine milk. The increase of calcium content might result from the cleavage of casein micelles under pressure, releasing some colloidal calcium phosphate bound to casein. In contrast, pasteurization and pasteurization-HHP treatment caused a slight decrease in the content of calcium, which might be due to the denaturation of whey protein under the action of heat treatment and deposition on the surface of casein micelles, causing protein molecules to aggregate, making it difficult to release colloidal calcium phosphate bound to casein, even if under pressure [7,51].

As shown in Figure 2B, the content of calcium in HHP, pasteurization and pasteurization-HHP-treated samples increased from 1052.75, 1011.00 and 1001.00 mg/kg to 1164.50, 1212.25 and 1150.75 mg/kg at day 3, respectively. However, the calcium content in these samples did not change significantly from day 3 to 30 (*p* > 0.05), fluctuating within a certain range. Nassar et al. [50] found that the content of calcium in caprine milk treated with HHP of 500 MPa/25 min during storage at 4 °C for 1–4 days also increased significantly (*p* < 0.05), and pointed out that this phenomenon could be attributed to calcium dissolution in the storage process.

#### 3.3.3. β-LG

After HHP, pasteurization and pasteurization-HHP treatments, the content of β-LG in milk was reduced from 592.61 μg/mL to 456.51, 422.82 and 355.37 μg/mL, respectively (Figure 2C). Under pressure, β-LG unfolded and aggregated through thiol-disulfide exchange and hydrophobic interactions, and this structural change caused β-LG to become more susceptible to protease hydrolysis, resulting in a decrease in β-LG content under HHP [52,53,54]. However, both pasteurization- and pasteurization-HHP- treatment caused more severe reduction in milk β-LG content. This result could probably be attributed to the fact that, in the process of pasteurization, the unfolding of the protein structure destroyed the tertiary structure and broke the disulfide bond, which made it lose its natural activity and become more likely to be hydrolyzed by protease under HHP [55,56,57].

As shown in Figure 2C, the content of β-LG in milk treated by HHP, pasteurization and pasteurization-HHP decreased from 456.51, 422.82 and 355.37 μg/mL to 307.26, 270.56 and 227.76 μg/mL during storage, respectively. Due to the hydrophobic interaction, it is difficult for denatured β-LG to re-polymerize at low temperature [58]. Meanwhile, the denatured β-LG had been increasing over time [59] and was easily hydrolyzed by proteases [60], resulting in the reduction of β-LG during storage. During storage, compared with HHP-treated milk, the decrease of β-LG content in pasteurization-treated and pasteurization-HHP-treated milk was slighter. It was speculated that more proteases were inactivated by heat treatment, so that hydrolysis of β-LG was inhibited. Even so, the β-LG content of HHP-treated milk was consistently higher than that of pasteurization-treated and pasteurization-HHP-treated milk during storage.

### 3.4. Alkaline Phosphatase Activity

**Figure 3:**
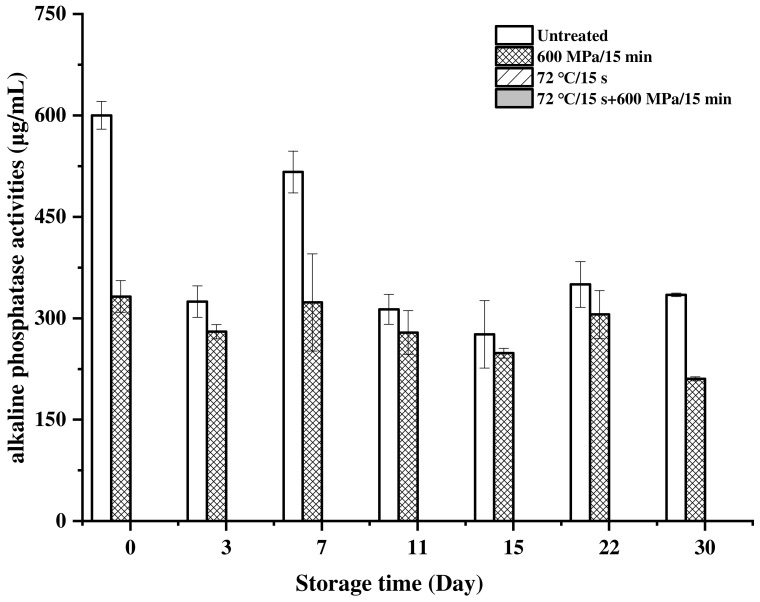
Changes in alkaline phosphatase activities of milk processed with HHP (600 MPa/15 min), pasteurization (72 °C/15 s) and pasteurization-HHP (72 °C/15 s + 600 MPa/15 min) during storage at 4 °C.

As a naturally occurring enzyme in milk, alkaline phosphatase is usually used to determine the intensity of heat treatment [61,62]. As shown in Figure 3, the initial alkaline phosphatase activity in the untreated milk was 600.13 μg/mL and all treatments resulted in a significant inactivation of alkaline phosphatase (*p* < 0.05). HHP treatment inactivated about 45% of the alkaline phosphatase activity in milk and reduced it to 332.19 μg/mL. In addition, the activity in HHP-treated milk decreased to 210.29 μg/mL at day 30. Alkaline phosphatase is an enzyme involved in the fat globule membrane; therefore, it is speculated that the decrease in alkaline phosphatase activity in milk could be related to the decrease in fat (Figure 2A) during storage [63]. However, no activity was detected in the pasteurization- and pasteurization-HHP-treated milk after treatments and during the storage at 4 °C. These results confirmed a lower degree of heat treatment in HHP-treated milk compared with pasteurization-treated, which might be conducive to maintaining nutritional quality.

### 3.5. Viscosity

**Figure 4:**
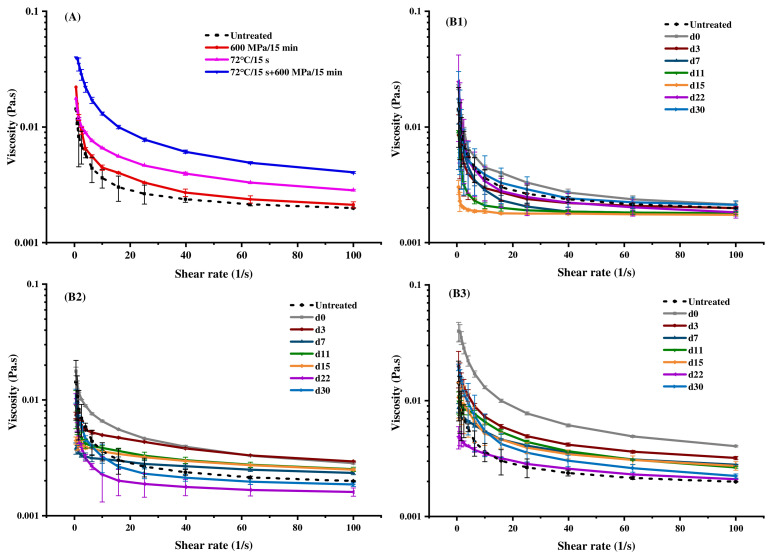
Effects of HHP (600 MPa/15 min), pasteurization (72 °C/15 s) and pasteurization-HHP (72 °C/15 s + 600 MPa/15 min) treatments on the viscosity of milk. ((**A**) viscosity of milk after treatments; (**B1**–**B3**) viscosity of HHP-, pasteurization- and pasteurization-HHP-treated milk during storage, respectively).

As shown in Figure 4A, with an increasing shear rate, the viscosity of milk declined and presented the shear-thinning phenomenon, which was consistent with the earlier research results [34]. Although all treatments increased milk viscosity, the viscosity of HHP-treated milk was closest to that of untreated milk, which means that HHP had minimal effect on the original rheological properties of milk. Pressure changed the natural volume of protein, leading to the dissociation of casein micelles and the unfolding of the structure of whey protein, especially β-LG [64], so that caseinate and whey protein were in close contact, forming a porous and dense chain polymer network, which was conducive to combination with water molecules and increased the viscosity of milk [65,66]. Likewise, heat treatment could also cause the unfolding of milk protein structure, increasing milk viscosity [67], and this effect was more significant than that of HHP treatment. Consequently, for pasteurization-HHP-treated milk, heat treatment caused the unfolded structure of milk protein, and the subsequent HHP treatment was more conducive to the aggregation of the protein and the formation of the gel network, making it exhibit the highest viscosity.

As shown in Figure 4B, all samples still reflected the characteristics of pseudoplastic fluid during storage. However, with the extension of storage time, the characteristics of the pseudoplastic fluid and the shear-thinning phenomenon became less obvious. Moreover, the viscosity of all samples showed a decreasing trend during storage. Since milk viscosity has a strong positive correlation with the contents of protein and fat [36,50,68], it was speculated that the decrease of milk viscosity during storage was mainly related to the decrease of fat content (Figure 2A). Compared with the HHP-treated milk, the viscosity of pasteurization-HHP-treated milk had a more significant change, which also corresponded to the reduction in degree of fat content.

### 3.6. Particle Size

**Figure 5:**
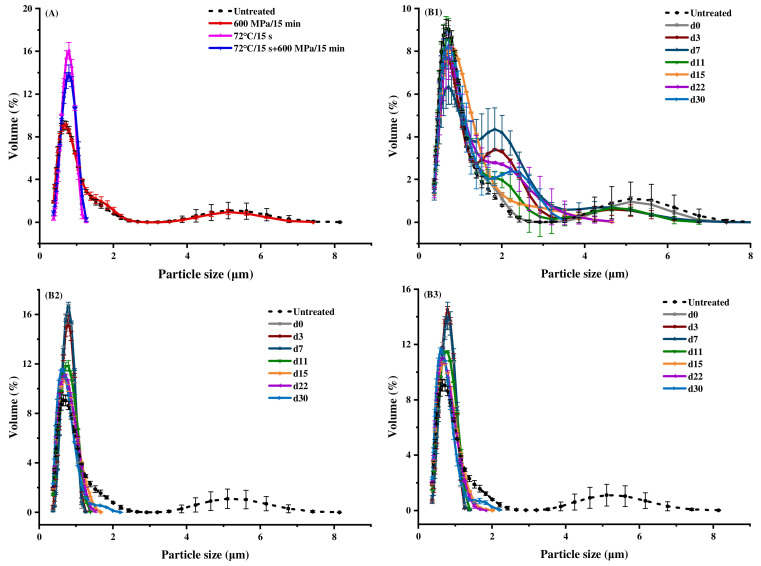
Effects of HHP (600 MPa/15 min), pasteurization (72 °C/15 s) and pasteurization-HHP (72 °C/15 s + 600 MPa/15 min) treatments on particle size distribution of milk ((**A**) particle size distribution after treatments; (**B1**–**B3**) particle size distribution of HHP-, pasteurization- and pasteurization-HHP-treated milk during storage, respectively).

The particle size distribution of untreated milk ranged from 0.37 to 8.14 μm, with peaks at 0.65 and 5.11 μm (Figure 5A). The shape of the particle size distribution curve was not changed after HHP treatment, while it changed greatly after pasteurization and pasteurization-HHP treatments, in which the maximum particle size was reduced from 8.14 μm to 1.38 μm, the peak value was only at 0.79 μm, and the distribution was more concentrated. Correspondingly, as shown in Table 3, the D(4,3) and D(3,2) of untreated milk were 4.98 and 3.71 μm, respectively, and there was no significant difference in D(4,3) and D(3,2) after HHP treatments (*p* > 0.05), while pasteurization and pasteurization-HHP treatments resulted in a significant reduction (*p* < 0.05). Ye et al. [69] found that the treatment of 100–800 MPa/30 min would not affect the particle size in the study of whole milk treated by HHP, which was consistent with our results. Huppertz et al. [42] pointed out that the change in the particle size was mainly caused by the fat particle size, and, therefore, since there was no pronounced effect on the fat particle size after HHP processing occurred, no significant difference was observed in the particle size. In contrast, pasteurization and pasteurization-HHP treatments caused a significant decrease in the particle size of milk (*p* < 0.05), which was probably because heat treatment destroyed the fat globule membrane, resulting in the dispersion of fat globule droplets and reduction in particle size [70].

During storage, the D(4,3) and D(3,2) of the particle size of the HHP-treated milk significantly reduced (*p* < 0.05), but that of the pasteurization- and pasteurization-HHP- treated milk significantly increased (*p* < 0.05) (Table 3). A consistent conclusion could also be drawn from the particle size distribution diagram (Figure 5B). The maximum particle size of HHP-treated milk was reduced from 8.14 μm to 3.51 μm, and the distribution was more concentrated (Figure 5B). However, the maximum particle size of pasteurization- and pasteurization-HHP-treated milk increased from 1.26 μm to 2.20 μm, the peak around 0.75 μm was broadened and the distribution was more dispersed. The increase of the particle size of pasteurization- and pasteurization-HHP-treated milk was probably due to the high intensity of heat treatment, which endowed the milk particles with higher energy, leading to Brownish motion, collision contact and droplet aggregation of milk particles during storage [71]. This may be another reason why the viscosity of pasteurization-HHP-treated milk was always higher than that of HHP-treated milk (Figure 4).

### 3.7. Sensory Evaluation

Sensory qualities among HHP-, pasteurization- and pasteurization-HHP-treated milk were determined after processing and during storage (Table 4). Taking into account the low safety and a large number of microorganisms, the taste of untreated milk was not scored.

After treatments, compared with untreated milk, the HHP-treated milk was significantly lower (*p* < 0.05), while pasteurization- and pasteurization-HHP-treated milk had no significant difference in the color score (*p* > 0.05). The reason for the low color score of HHP-treated milk was mainly due to the decrease in milk brightness caused by HHP treatment, which resulted in a pronounced increase in Δ*E*. The milk was homogenized in advance, so that no obvious floating fat and stratification were observed after different treatments, so, in consequence, there was no significant difference in the tissue state score (*p* < 0.05).

Moreover, after treatments, the flavor scores of all samples were significantly improved (*p* < 0.05), which may be related to the changes in flavor substances during the processing. HHP caused the minimum change in the volatile composition of milk, heat treatment at high temperature promoted the formation of both aldehydes and methyl ketones, whereas the combination of high pressure and temperature favored the formation of aldehydes [72]. The taste of pasteurization- and pasteurization-HHP-treated milk was significantly better than HHP-treated milk (*p* < 0.05), which was probably because pasteurization- and pasteurization-HHP-treated milk had smaller particle size (Figure 5) and higher viscosity (Figure 4) compared with HHP-treated milk, leading to a more delicate and mellow taste. It must be noted that people probably have become accustomed to the taste and flavor of heat-processed milk, which leads to a decline in the acceptance of HHP-treated milk, that is similar to the original milk in quality, resulting in the low sensory score of HHP-treated milk.

The sensory score variation of milk processed with different treatments during storage is shown in Table 4. Since the number of microorganisms exceeded the standard after the 11th day and the safety of milk could not be guaranteed, the taste could not be scored. Therefore, only the sensory evaluation of the first 11 days of storage was carried out. As shown in Table 4, the sensory scores of all treated samples gradually decreased during the 11 days, and the degree of decrease exhibited significance until day 11. In addition, the total sensory score of HHP-treated milk was always lower than that of pasteurization- and pasteurization-HHP-treated milk during storage, while there was no significant difference between the latter two. It can be seen that the sensory acceptance of HHP milk was still not high.

### 3.8. Principal Component Analysis (PCA)

**Figure 6:**
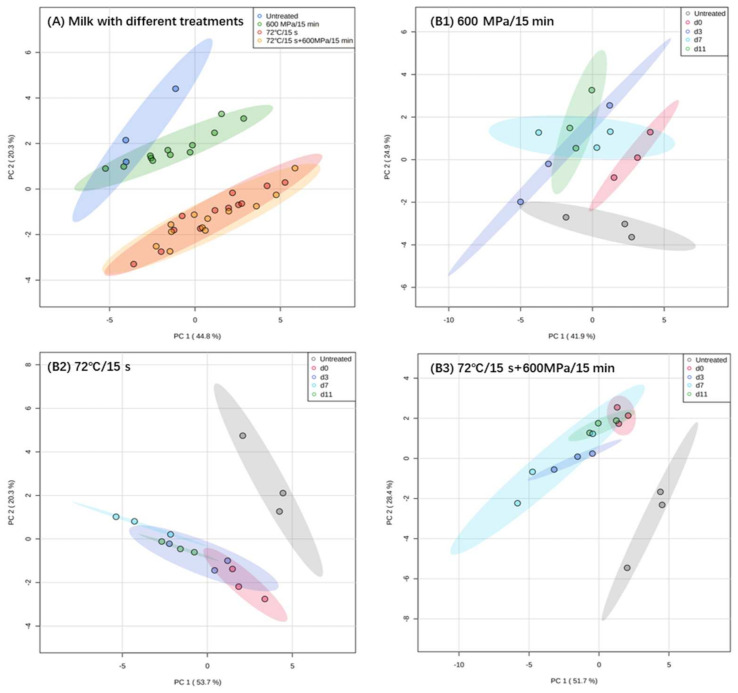
PCA of the integrated quality of untreated, HHP-treated (600 MPa/15 min), pasteurization-treated (72 °C/15 s) and pasteurization−HHP-treated (72 °C/15 s + 600 MPa/15 min) milk. ((**A**) quality of milk processed with different treatments; (**B1**–**B3**) changes in milk processed with one treatment during storage).

Principal Component Analysis (PCA) is commonly used for data dimensionality reduction. In this study, 18 indicators, including physicochemical characteristics, nutritional quality, and sensory evaluation of milk, were analyzed by PCA for data dimensionality reduction. Figure 6A presents the PCAs of milk processed with different treatments. Apparently, the quality of HHP-treated milk can be clearly distinguished from pasteurization- and pasteurization-HHP-treated milk. Among the milk processed with different treatments, only the HHP treated milk was similar to the untreated milk, which indicated that HHP treatment had less impact on the quality of the milk.

Furthermore, as shown in Figure 6B1, the clustering of HHP-treated milk at different storage times showed a wide range of overlap, and suggested that there were no significant quality changes during storage. In contrast, the quality of pasteurization-treated milk quality changed more significantly (Figure 6B2). In general, the comprehensive quality of HHP milk was closer to that of untreated milk, and it performed well in storage stability.

## 4. Conclusions

Compared to pasteurization, HHP treated milk exhibited an excellent inactivation effect after processing and better microbiological stability during 30 days of storage at 4 °C. None of the three treatments caused significant damage to the nutrient content of milk, such as its protein, ash, fat and calcium, while HHP-treated milk exhibited better stability in nutritional quality during storage. Furthermore, HHP treatment showed lower heat treatment intensity and less impact on pH, viscosity and particle size, but it performed worse in terms of brightness and sensory quality. PCA showed that the quality of HHP-treated milk differed from pasteurization- and pasteurization-HHP-treated milk and was more similar to the natural untreated milk.

In sum, compared with pasteurization and pasteurization-HHP, HHP better improved microbiological stability, extended shelf life, and maintained nutrient stability of milk, but did not perform well in sensory evaluation. If consumers’ acceptance of the original flavor of milk could be correctly guided, the prospects of HHP-treated milk are broader.

## Figures and Tables

**Table 1:** The counts of total aerobic bacteria (TAB) and yeasts and molds (Y&M) in the milk after HHP treatments with different pressures (400, 500 and 600 MPa) and processing times (2.5, 5, 7.5, 10 and 15 min) at 25 °C, compared to the untreated milk.

	Processing Time (min)	TAB (lgCFU/mL)	Y&M (lgCFU/mL)
Untreated		4.19 ± 0.08 ^m^	1.82 ± 0.16 ^a^
400 MPa	2.5	3.84 ± 0.09 ^l^	0.09 ± 0.12 ^b^
	5	3.38 ± 0.09 ^k^	ND
	7.5	2.90 ± 0.01 ^j^	ND
	10	2.85 ± 0.10 ^ij^	ND
	15	2.81 ± 0.09 ^ij^	ND
500 MPa	2.5	2.74 ± 0.05 ^hi^	ND
	5	2.65 ± 0.07 ^gh^	ND
	7.5	2.57 ± 0.08 ^fg^	ND
	10	2.49 ± 0.01 ^ef^	ND
	15	2.41 ± 0.02 ^e^	ND
600 MPa	2.5	2.20 ± 0.04 ^d^	ND
	5	1.99 ± 0.09 ^c^	ND
	7.5	1.41 ± 0.09 ^b^	ND
	10	1.31 ± 0.27 ^b^	ND
	15	1.12 ± 0.06 ^a^	ND

ND, no detected (detection limit <1 CFU/mL). Values in the same column with different superscript letters indicate significant differences (*p* < 0.05).

**Table 2:** Changes in pH, turbidity and color parameters of milk processed with HHP (600 MPa/15 min), pasteurization (72 °C/15 s) and pasteurization-HHP (72 °C/15 s + 600 MPa/15 min) during storage at 4 °C.

		Day						
		0	3	7	11	15	22	30
pH	Untreated	6.45 ± 0.15 ^Ac^	-	-	-	-	-	-
	600 MPa/15 min	6.45 ± 0.07 ^Ab^	6.36 ± 0.04 ^ab^	6.36 ± 0.04 ^ab^	6.26 ± 0.13 ^ab^	6.31 ± 0.11 ^ab^	6.25 ± 0.02 ^ab^	6.26 ± 0.07 ^ab^
	72 °C/15 s	6.68 ± 0.01 ^Bd^	6.66 ± 0.01 ^d^	6.64 ± 0.01 ^cd^	6.66 ± 0.01 ^d^	6.59 ± 0.01 ^c^	6.43 ± 0.01 ^b^	6.18 ± 0.07 ^a^
	72 °C/15 s + 600MPa/15 min	6.69 ± 0.01 ^Be^	6.64 ± 0.02 ^c^	6.65 ± 0.01 ^cd^	6.68 ± 0.02 ^de^	6.69 ± 0.01 ^e^	6.56 ± 0.01 ^b^	6.39 ± 0.01 ^a^
Turbidity (NTU)	Untreated	384.10 ± 11.74 ^Bb^	-	-	-	-	-	-
	600 MPa/15 min	326.10 ± 18.67 ^Aab^	324.60 ± 15.70 ^ab^	305.40 ± 23.33 ^a^	309.23 ± 17.93 ^a^	356.00 ± 4.24 ^b^	328.75 ± 22.41 ^ab^	330.00 ± 2.12 ^ab^
	72 °C/15 s	422.30 ± 43.70 ^Bab^	408.5 ± 52.18 ^ab^	388.70 ± 15.70 ^ab^	383.00 ± 5.09 ^ab^	448.70 ± 1.27 ^b^	400.80 ± 50.35 ^ab^	362.00 ± 2.55 ^a^
	72 °C/15 s + 600 MPa/15 min	325.45 ± 45.75 ^Aab^	341.05 ± 4.17 ^ab^	297.00 ± 2.12 ^ab^	293.40 ± 7.21 ^ab^	335.95 ± 8.84 ^b^	327.9 ± 0.85 ^ab^	313.20 ± 0.42 ^a^
L^*^	Untreated	82.76 ± 0.27 ^B^	-	-	-	-	-	-
	600 MPa/15 min	80.79 ± 0.39 ^Aab^	80.91 ± 0.65 ^b^	80.36 ± 0.68 ^ab^	80.53 ± 0.61 ^ab^	80.85 ± 0.93 ^ab^	79.90 ± 0.10 ^a^	80.24 ± 0.21 ^ab^
	72 °C/15 s	82.64 ± 0.94 ^Bbc^	81.945 ± 0.57 ^ab^	82.80 ± 0.22 ^c^	83.22 ± 0.23 ^cd^	83.77 ± 0.16 ^d^	82.57 ± 0.34 ^bc^	81.43 ± 0.14 ^a^
	72 °C/15 s + 600 MPa/15 min	81.26 ± 0.66 ^Abc^	80.98 ± 0.44 ^b^	81.64 ± 0.14 ^cd^	81.81 ± 0.24 ^cd^	82.17 ± 0.33 ^d^	80.84 ± 0.67 ^b^	80.04 ± 0.03 ^a^
a^*^	Untreated	−1.51 ± 0.06 ^B^	-	-	-	-	-	-
	600 MPa/15 min	−1.71 ± 0.05 ^Abc^	−1.62 ± 0.40 ^c^	−1.88 ± 0.03 ^b^	−2.13 ± 0.07 ^a^	−1.69 ± 0.06 ^bc^	−1.79 ± 0.04 ^bc^	−1.19 ± 0.04 ^d^
	72 °C/15 s	−1.49 ± 0.06 ^Bb^	−1.27 ± 0.05 ^c^	−1.70 ± 0.14 ^a^	−1.77 ± 0.06 ^a^	−1.51 ± 0.05 ^b^	−1.43 ± 0.06 ^b^	−1.18 ± 0.036 ^c^
	72 °C/15 s + 600 MPa/15 min	−1.73 ± 0.05 ^Ab^	−1.56 ± 0.07 ^c^	−1.87 ± 0.11 ^a^	−1.93 ± 0.04 ^a^	−1.72 ± 0.043 ^b^	−1.64 ± 0.03 ^bc^	−1.31 ± 0.06 ^d^
b^*^	Untreated	1.95 ± 0.17^B^	-	-	-	-	-	-
	600 MPa/15 min	1.36 ± 0.31 ^Abc^	1.46 ± 0.32 ^bc^	1.48 ± 0.35 ^bc^	1.61 ± 0.25 ^c^	1.06 ± 0.36 ^ab^	0.71 ± 0.32 ^a^	3.08 ± 0.10 ^d^
	72 °C/15 s	2.20 ± 0.16 ^Bbc^	2.32 ± 0.44 ^c^	1.27 ± 0.14 ^a^	1.86 ± 0.35 ^b^	2.18 ± 0.08 ^bc^	2.14 ± 0.14 ^bc^	3.11 ± 0.06 ^d^
	72 °C/15 s + 600 MPa/15 min	1.46 ± 0.21 ^Aa^	1.65 ± 0.20 ^a^	1.68 ± 0.13 ^a^	1.59 ± 0.18 ^a^	1.45 ± 0.06 ^a^	1.49 ± 0.26 ^a^	3.16 ± 0.05 ^b^
Δ*E*	Untreated	0	-	-	-	-	-	-
	600 MPa/15 min	4.24	3.66	6.1	5.47	4.45	9.79	7.72
	72 °C/15 s	0.08	0.86	0.50	0.29	1.07	0.76	3.22
	72 °C/15 s + 600 MPa/15 min	2.52	3.25	1.44	1.20	0.65	3.91	8.88

-, not tested. Values in the same column with different superscript capital letters indicate significant differences between the samples after different treatments (*p* < 0.05). Values in the same line with different superscript lowercase letters indicate significant changes of one sample during storage (*p* < 0.05).

**Table 3:** Changes in D(4,3) and D(3,2) of milk processed with HHP (600 MPa/15 min), pasteurization (72 °C/15 s) and pasteurization-HHP (72 °C/15 s + 600 MPa/15 min) during storage at 4 °C.

	Day	Untreated	600 MPa/15 min	72 °C/15 s	72 °C/15 s + 600 MPa/15 min
D(4,3) (nm)	0	4.98 ± 0.54 ^Bb^	4.82 ± 0.16 ^Bc^	0.84 ± 0.01 ^Aa^	0.87 ± 0.01 ^Aa^
	3	-	3.75 ± 0.28 ^b^	0.85 ± 0.01 ^a^	0.87 ± 0.01 ^a^
	7	-	3.70 ± 0.06 ^b^	0.85 ± 0.05 ^a^	0.88 ± 0.01 ^ab^
	11	-	4.02 ± 0.17 ^b^	0.89 ± 0.01 ^b^	0.90 ± 0.01 ^b^
	15	-	2.17 ± 0.16 ^a^	0.94 ± 0.02 ^d^	0.99 ± 0.02 ^d^
	22	-	2.25 ± 0.49 ^a^	0.91 ± 0.01 ^c^	0.94 ± 0.01 ^c^
	30	-	2.12 ± 0.17 ^a^	1.02 ± 0.01 ^e^	1.07 ± 0.01 ^e^
D(3,2) (nm)	0	3.71 ± 1.02 ^Ba^	3.52 ± 0.30 ^Bc^	0.81 ± 0.01 ^Aa^	0.83 ± 0.01 ^Aa^
	3	-	2.71 ± 0.26 ^b^	0.82 ± 0.01 ^a^	0.83 ± 0.01 ^a^
	7	-	2.82 ± 0.11 ^b^	0.82 ± 0.04 ^a^	0.84 ± 0.06 ^ab^
	11	-	2.91 ± 0.22 ^b^	0.84 ± 0.01 ^b^	0.85 ± 0.01 ^bc^
	15	-	1.69 ± 0.09 ^a^	0.87 ± 0.01 ^c^	0.90 ± 0.01 ^d^
	22	-	1.87 ± 0.39 ^a^	0.85 ± 0.01 ^b^	0.86 ± 0.01 ^c^
	30	-	1.77 ± 0.12 ^a^	0.89 ± 0.01 ^c^	0.91 ± 0.01 ^d^

-, not tested. Values in the same line with different superscript capital letters indicate significant differences between the samples after different treatments *(p* < 0.05). Values in the same column with different superscript lowercase letters indicate significant changes of one sample during storage *(p <* 0.05).

**Table 4:** Changes in the sensory score of milk processed with HHP (600 MPa/15 min), pasteurization (72 °C/15 s) and pasteurization-HHP (72 °C/15 s + 600 MPa/15 min) during storage at 4 °C.

	Day	Untreated	600 MPa/15 Min	72°C/15 s	72 °C/15 s + 600 MPa/15 min
Taste	0	-	20.57 ± 8.18 ^Ab^	33.43 ± 4.31 ^Bb^	35.43 ± 3.96 ^Bb^
	3	-	19.60 ± 8.84 ^b^	32.93 ± 3.73 ^b^	34.27 ± 4.68 ^ab^
	7	-	17.86 ± 8.16 ^a^	31.87 ± 3.74 ^ab^	33.20 ± 4.57 ^ab^
	11	-	16.53 ± 6.06 ^a^	29.26 ± 4.77 ^a^	31.20 ± 5.21 ^a^
Color	0	16.36 ± 1.91 ^Bb^	14.00 ± 2.21 ^Ab^	16.29 ± 2.27 ^Ba^	15.07 ± 1.86 ^ABa^
	3		13.47 ± 2.53 ^ab^	16.13 ± 2.41 ^a^	15.00 ± 2.33 ^a^
	7		13.78 ± 1.76 ^ab^	15.67 ± 2.77 ^a^	14.73 ± 1.87 ^a^
	11		13.20 ± 2.42 ^a^	14.93 ± 2.96 ^a^	13.46 ± 2.45 ^a^
Organizational status	0	17.36 ± 2.13 ^Aa^	17.71 ± 2.05 ^Aa^	18.00 ± 1.41 ^Aa^	18.14 ± 1.70 ^Aa^
	3		17.00 ± 2.78 ^a^	17.40 ± 1.86 ^a^	17.93 ± 1.62 ^a^
	7		16.57 ± 2.21 ^a^	17.20 ± 1.74 ^a^	17.27 ± 1.33 ^a^
	11		15.46 ± 2.38 ^a^	17.07 ± 1.62 ^a^	16.67 ± 2.02 ^a^
Flavor	0	11.86 ± 2.93 ^Ac^	14.42 ± 3.23 ^Ab^	15.71 ± 3.10 ^Aa^	16.21 ± 1.84 ^Aa^
	3		14.53 ± 2.82 ^b^	15.20 ± 3.12 ^a^	15.87 ± 2.20 ^a^
	7		13.71 ± 1.94 ^a^	14.93 ± 2.74 ^a^	15.60 ± 1.80 ^a^
	11		13.33 ± 2.19 ^a^	14.93 ± 2.08 ^a^	15.27 ± 2.52 ^a^
Total score	0	-	66.71 ± 9.48 ^Ab^	83.43 ± 8.75 ^Bb^	84.86 ± 5.68 ^Bb^
	3	-	64.60 ± 9.94 ^b^	81.67 ± 7.43 ^b^	83.01 ± 6.09 ^b^
	7	-	61.47 ± 9.62 ^b^	79.67 ± 7.09 ^ab^	80.80 ± 6.85 ^b^
	11	-	58.53 ± 5.69 ^a^	76.20 ± 7.01 ^a^	76.60 ± 7.42 ^a^

Different lowercase letters in the same column represented significant differences in the scores of the milk processed with the same treatment during storage (*p* < 0.05). Different capital letters on the same line indicated significant differences in the scores of the milk processed with different treatments (*p* < 0.05). -, not tested.

## Data Availability

The raw data supporting the conclusion of this article will be made available by the authors, without undue reservation.

## Supplementary Materials

The following supporting information can be downloaded at: https://www.mdpi.com/article/10.3390/foods11182837/s1. Figure S1: Changes in furosine content of milk during storage; Figure S2: Changes in protein (A) and ash (B) content of milk during storage; Table S1: Standard score sheet for the sensory evaluation of milk.
