# Supplementary material for: Comparison of the Effects of High Hydrostatic Pressure and Pasteurization on Quality of Milk during Storage
Source: Foods. 2022 Sep 14;11(18):2837. doi: 10.3390/foods11182837 (PMC9498420; doi:10.3390/foods11182837)
Supplement: Supplementary file 1 [file foods-11-02837-s001.zip › foods-1849291-supplementary.pdf]

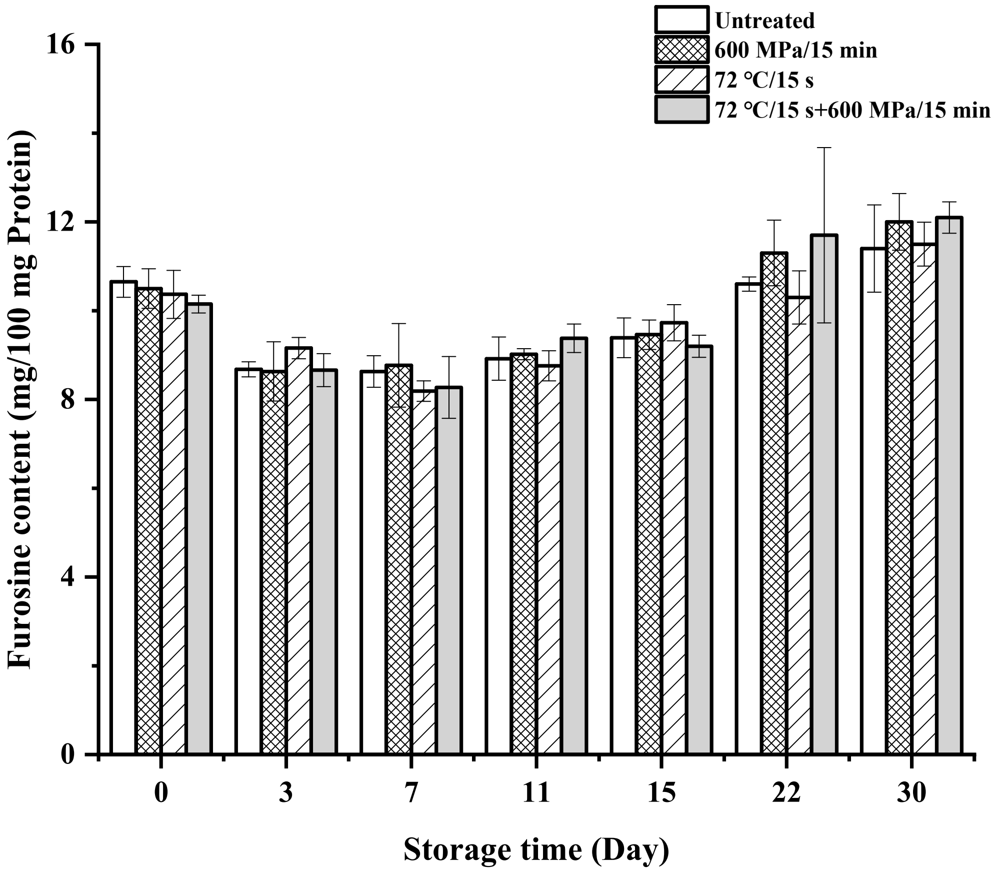

Figure S1. Changes in furosine content of milk processed with HHP (600 MPa/15 min), pasteurization (72°C/15 s) and pasteurization-HHP (72°C/15 s+600 MPa/15 min) during storage at 4°C.

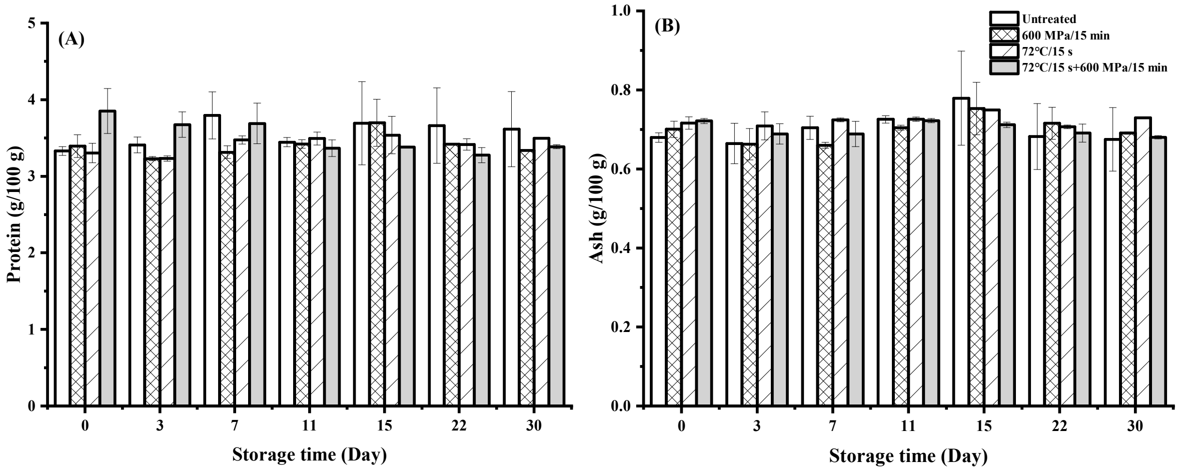

Figure S2. Changes in protein (A) and ash (B) content of milk processed with HHP (600 MPa/15 min), pasteurization (72°C/15 s) and pasteurization-HHP (72°C/15 s+600 MPa/15 min) during storage at 4°C.

Table S1 Standard score sheet for the sensory evaluation of milk

Indicators	Standard	Score
Taste	Soft, delicate and sweet, with milky fragrance	30-40
	Relatively delicate, slightly sweet with no mutton	16-29
	Heavy mutton	1-15
Color	Milky white	16-20
	Slightly yellow	11-15
	Yellow	1-10
Organizational status	Uniform with no fat floating	16-20
	Uniform with slight delamination	11-15
	Obvious stratification, precipitation and clot	1-10
Flavor	Strong and durable milky fragrance	16-20
	Light and unsustainable milky fragrance	11-15
	No milk fragrance, heavy peculiar smell	1-10
